# Exploring immune related gene signatures and mechanisms linking non alcoholic fatty liver disease to atrial fibrillation through transcriptome data analysis

**DOI:** 10.1038/s41598-023-44884-z

**Published:** 2023-10-16

**Authors:** Keke Wu, Jiayi Zhu, Yingxu Ma, Yong Zhou, Qiuzhen Lin, Tao Tu, Qiming Liu

**Affiliations:** 1 Department of Cardiovascular Medicine, The Second Xiangya Hospital, Central South University, 139 Renmin Middle Road, Changsha, 410011 Hunan People’s Republic of China; 2 Research Institute of Blood Lipid and Atherosclerosis, Central South University, Changsha, People’s Republic of China; 3 Modern Cardiovascular Disease Clinical Technology Research Center of Hunan Province, Changsha, People’s Republic of China; 4 Cardiovascular Disease Research Center of Hunan Province, Changsha, 410011 Hunan People’s Republic of China

**Keywords:** Bioinformatics, Atrial fibrillation

## Abstract

Atrial fibrillation (AF) and related cardiovascular complications pose a heavy burden to patients and society. Mounting evidence suggests a close association between nonalcoholic fatty liver disease (NAFLD) and AF. NAFLD and AF transcriptomic datasets were obtained from GEO database and analyzed using several bioinformatics approaches. We established a NAFLD-AF associated gene diagnostic signature (NAGDS) using protein–protein interaction analysis and machine learning, which was further quantified through RT-qPCR. Potential miRNA targeting NAGDS were predicted. Gene modules highly correlated with NAFLD liver pathology or AF occurrence were identified by WGCNA. Enrichment analysis of the overlapped genes from key module revealed that T-cell activation plays essential roles in NAFLD and AF, which was further confirmed by immune infiltration. Furthermore, an integrated SVM-RFE and LASSO algorithm was used to identify CCL4, CD48, ITGB2, and RNASE6 as NAGDS, all of which were found to be upregulated in NAFLD and AF mouse tissues. Patients with higher NAGDS showed augmented T cell and macrophage immunity, more advanced liver pathological characteristics, and prolonged AF duration. Additionally, hsa-miR-26a-5p played a central role in the regulation of NAGDS. Our findings highlight the central role of T-cell immune response in linking NAFLD to AF, and established an accurate NAGDS diagnostic model, which could serve as potential targets for immunoregulatory therapy.

## Introduction

Atrial fibrillation (AF) is the most common sustained cardiac arrhythmia, with a current prevalence ranging between 2 and 4%. However, this is expected to increase 2.3-fold due to the aging population and intensified screening for undiagnosed AF^1^. The rising prevalence of AF can be attributed to numerous risk factors, including aging, genetic predisposition, obesity, smoking, diabetes (DM), and inflammatory diseases^2^. Nevertheless, not all AF cases can be explained by the aforementioned risk factors, underscoring the importance of identifying new triggers.

Nonalcoholic fatty liver disease (NAFLD), characterized by excessive hepatic fat accumulation, is the most common cause of chronic liver disease in clinical practice^3^. The global prevalence of NAFLD accounted for about 25% in 2018^4^. The number of NAFLD patients even exceeds the population of obesity (650 million) coupled with DM (400 million), the 2 key risk factors for AF^5^. To date, compelling evidence also indicates a close association between the presence of NAFLD and an increased risk of AF^6–8^. There must be predisposing factors in NAFLD patients that make them more susceptible to AF. It is well-known that NAFLD is a disease related to systemic inflammation and oxidative stress, which may trigger arrhythmogenic injury (i.e., structure, electrical, and autonomic remodeling) to the heart^6,9,10^. However, the direct evidence is little, and fails to reveal the mechanism of AF secondary to NAFLD at the genetic level.

To explore the “trigger point” between NAFLD and AF, we used weighted gene co-expression network analysis (WGCNA) to identify correlated and shared gene clusters in NAFLD and AF. Our analysis revealed the presence of T cell activation-associated genes within modules most closely related to NAFLD or AF, and there were differences in immune infiltration between patients and normal individuals, particularly evident in T cells. These results indicate mechanisms of AF secondary to NAFLD may be associated with T cell-mediated immunity. In addition, we established a NAFLD-AF gene diagnostic signature (NAGDS) for supplementary diagnosis of NAFLD and AF by protein–protein interaction (PPI) analysis and machine learning, and explored the potential diagnostic and therapeutic values underlying NAGDS.

## Materials and methods

### Data collection and preprocessing

**Figure 1:**
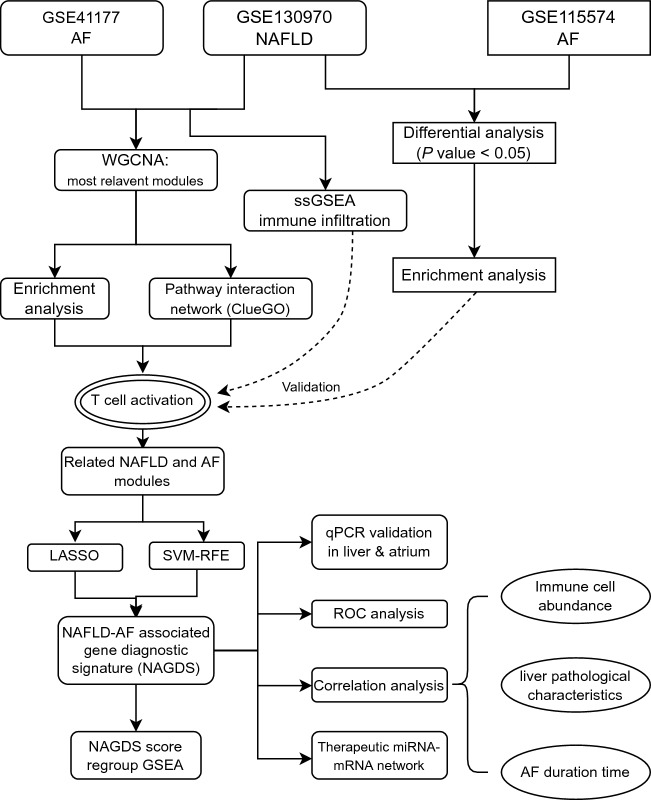
Study workflow. AF, atrial fibrillation; GSEA, gene set enrichment analysis; LASSO, least absolute shrinkage and selection operator; NAFLD, nonalcoholic fatty liver disease; ROC, receiver operating characteristic; ssGSEA, single-sample gene set enrichment analysis; SVM-RFE, support vector machine recursive feature elimination; WGCNA, weighted gene co-expression network analysis.

The overall workflow of this study is shown in Fig. 1. Two NAFLD and four AF datasets were downloaded from the GEO database (http://www.ncbi.nlm.nih.gov/geo). Raw probe matrix of GSE41177 (named as AF dataset), GSE115574, GSE14975, and GSE63067 were annotated with GPL570 (Affymetrix Human Genome U133 Plus 2.0 Array) to generate gene expression matrix. Probes annotated to the same gene symbol were merged to their average levels. Samples from the left atrium-pulmonary vein junction were excluded in GSE41177. Gene expressions were normalized with the ‘limma’ (version 3.48.3) package in R^11^. Gene expression matrix of GSE130970 (named as NAFLD dataset), based on GPL16791 (Illumina HiSeq 2500), were downloaded as transcripts Per Kilobase per Million mapped reads (TPMs) and were subsequently transferred to gene symbol from Entrez id. Liver biopsy samples were categorized into two groups: those with a NAFLD activity score (NAS) ≥ 5, referred to as the NAFLD group, and those with NAS < 5, considered as the control group^12^. Gene expression data from GSE115574, GSE14975, and GSE63067 were used as validation datasets.

### WGCNA

Weighted gene co-expression network analysis was conducted in the NAFLD and AF datasets with the ‘WGCNA’ (version 1.70-3) package in R^13,14^. The power values were screened ranging from 1 to 20 using the ‘pickSoftThreshold’ function, and the minimum value of the degree of independence over 0.85 was used. Co-expression networks were constructed with the ‘blockwiseModules’ function with a merge cut-off height of 0.25. Genes inside the same module were considered significantly interconnected. Correlations between all modules and clinical traits were visualized with the ‘labeledHeatmap’ function of the ‘WGCNA’ package. We selected modules that exhibited the highest correlation with clinical characteristics for further analysis.

### Functional enrichment analysis

Gene Ontology (GO), Kyoto Encyclopedia of Genes and Genomes (KEGG), and the REACTOME pathway enrichment analysis were applied with the ‘clusterProfiler’ (version 4.0.5) and ‘ReactomePA’ (version 1.36.1) packages in R^15–18^. Biological processes, cell components, and molecular functions were enriched and investigated individually. The enriched terms with an adjusted *P* value of less than 0.05 were considered significant.

### PPI analysis

PPI networks were acquired from the STRING database (https://string-db.org/) and visualized using ‘Cytoscape’ software (version 3.9.1) with a confidence score greater than 0.4. Hub genes were screened with ‘MCODE’ (version 2.0.2)^19^ or ‘cytoHubba’ (version 0.1)^20^ plugins. ‘ClueGO’ (version 2.5.9)^21^ was used to visualize the GO terms network based on the similarity of their associated genes with GO terms fusion function and two-sided hypergeometric test. The degree of connectivity of each term’s connection was quantified with kappa statistics. Functional grouping of the GO terms was also based on the kappa score with a kappa score threshold of 0.4, and redundant groups with > 50% overlap were merged.

### Immune cell infiltration analysis

The gene expression matrix and a validated cell metagenes signature of 28 immune cell types (Table S3)^22,23^ were inputted to calculate immune cell abundance using the single-sample gene set enrichment analysis (ssGSEA) method of ‘GSVA’ (version 1.40.1) package in R^24^. Cell subsets were compared in each group using the Mann–Whitney *U*-test and visualized via the ‘ggpubr’ (version 0.4.0) package in R. A *P* value of less than 0.05 were regarded as significant.

### Diagnostic signature screening and construction with machine learning

The support vector machine recursive feature elimination (SVM-RFE) algorithm was utilized in this study via the ‘rfe’ function of ‘caret’ (version 6.0-91) package in R with tenfold cross-validation for diagnostic gene signatures screening. SVM-RFE is a machine learning approach that recursively eliminates features and calculates each feature’s weight to screen out less important and meaningful information. We then used the least absolute shrinkage and selection operator (LASSO) regression with the ‘glmnet’ (version 4.1-4) package in R^25^ to further shrink the scope of selected features. Combined SVM-RFE and LASSO approach, genes with strong relevance to NAFLD and AF were identified with higher accuracy. Finally, NAGDS was constructed according to the expression of each significant gene and their respective coefficient from the LASSO regression: NAGDS score = Σ (significant gene expression) * (each gene’s coefficient).

### Correlation analysis between gene expression and clinical characteristics

Non-parametric Spearman’s correlation was calculated to determine the correlation between gene expression and liver pathological features in the NAFLD dataset, AF duration time in the AF dataset, and immune cell infiltration abundance in both datasets. Correlations were visualized using ‘ggplot2’ (version 3.3.5), ‘ggpubr’, and ‘ggExtra’ (version 0.9) packages in R.

### Predictive value of diagnostic signature

We used the receiver operating characteristic (ROC) curve with the ‘pROC’ (version 1.18.0) package in R to quantify the diagnostic effectiveness of individual genes or the overall NAGDS score^26^. GSE63067, a NAFLD dataset, and GSE14975, an AF dataset, were used as external validation sets to further verify the diagnostic accuracy of the diagnostic signature.

### Validation of immune activation in NAFLD and AF DEGs

Differentially expressed genes (DEGs) were screened using the ‘limma’ package with a *P*-value < 0.05 defined as significant. DEGs from the NAFLD dataset and GSE115574 were intersected to get overlapped DEGs.

### Animal models of NAFLD and AF

The animal study is reported in accordance with ARRIVE guidelines 2.0^27^. Male C57BL/6 mice, 8 weeks of age, each weighing 20–22 g, were purchased from the Institute of Laboratory Animal Science, Hunan SJA Laboratory Animal Co., Ltd. All animal experiments were under the approval of the Animal Care and Use Committee of Second Xiangya Hospital of Central South University and were performed in strict accordance with the recommendations in the Guide for the Care and Use of Laboratory Animals of the National Institutes of Health. Mice were housed in a specific-pathogen-free (SPF) environment with a regular 12/12 h day/night cycle, a temperature of 22 °C, and a humidity of 70%. After one week of adaptive feeding in the laboratory environment, the mice were then randomly divided into two groups: (1) control group (n = 6): animals treated with the standard chow diet; (2) High-fat diet (HFD) group (n = 6): animals given a high-fat Western diet (D12079B), and mice were allocated to six mice per cage. Confounders were not controlled in randomisation. Mice of each group were sacrificed at 12 weeks. Experimental animals were euthanized in advance if they developed irreversible diseases or health problems that would cause them severe pain or suffering. No animals were euthanized prior to the planned end of experiment. At the end of the experiments, all mice were euthanized by an intraperitoneal injection of sodium pentobarbital. Livers and atriums were immediately excised, thoroughly washed with ice-cold phosphate-buffered saline (PBS), and stored in liquid nitrogen until further use. For AF murine model, mice were randomly divided into two groups: (1) control group (n = 4): daily injection of saline through angular vein; (2) AF group (n = 4): daily injection of ACh (66 µg/kg) + CaCl_2_ (10 mg/kg) through angular vein, as previous reported^28^. The operator was blinded from the group allocation at all times. Mice were sacrificed at 3 weeks after the same euthanasia method, and hearts were harvested in the same methods mentioned above. An overall of twenty animals were used in this experiment, and the animal models and animal sample size were selected based on previous studies.

### Real-time quantitative PCR

Total mRNA was extracted from tissues with GeneJET RNA Purification Kit (Thermo Fisher Scientific). After determining RNA concentration and purity, cDNA was synthesized using the High-Capacity cDNA RT Kit with RNase Inhibitor (Thermo Fisher Scientific). RT-qPCR was conducted using the Applied Biosystems™ SYBR™ Green PCR Mix (Thermo Fisher Scientific) by BIO-RAD Real-Time PCR System. The expression level was quantized by 2^−ΔΔ^ CT mode. β-actin was used as reference for quantitative analysis. The primer sequences for RT-qPCR are listed in Supplementary Table S1.

### Gene set enrichment analysis between high and low NAGDS groups

Samples were regrouped by NAGDS score to the high NAGDS score group and low NAGDS score group. Genes were ranked by log_2_(Fold change) obtained from ‘limma’ differential analysis. Gene set enrichment analysis (GSEA) was applied between high and low NAGDS score groups in both NAFLD and AF datasets via the ‘gseGO’ function of ‘clusterProfiler’ package in R.

### Construction of therapeutic miRNA-mRNA network

Shared miRNAs that significantly changed in AF and NAFLD were downloaded from the HMDD database v3.2 (https://www.cuilab.cn/hmdd) that contained experiment-validated diseases-related miRNAs^29^. Potential miRNA-mRNA pairs were obtained from DIANA-TarBase v8 containing experimentally supported miRNA targets to construct a miRNA-mRNA network from NAGDS and NAFLD-AF shared miRNAs^30^.

### Statistical analysis

Statistical analysis was performed in R (version 4.1.2). Comparisons between the two groups were carried out with two-tailed Student’s *t-*test or Mann–Whitney *U*-test. The correlations among gene expression and other factors are assessed in Spearman’s correlation coefficients. The area under the ROC curve (AUC) represented diagnostic performance. For all assessments, a *P*-value < 0.05 was considered statistically significant, and the exact *P*-value were reported in results section.

## Results

### Identification of NAFLD and AF relevant key modules

**Figure 2:**
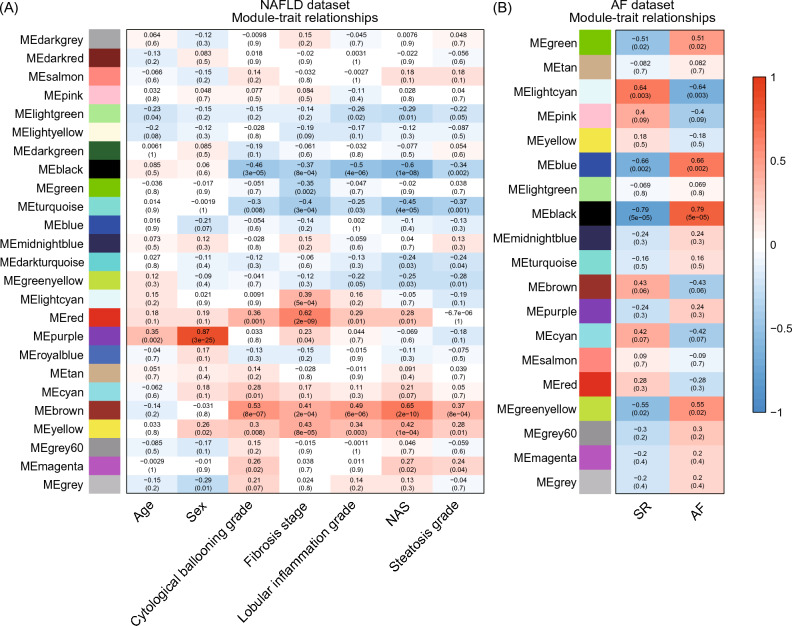
Correlation heatmaps of module-trait relationships from weighted co-expression network. (**A**) Correlation between pathological characteristics and module eigengenes of NAFLD dataset. (**B**). Correlation between AF status and module eigengenes of AF dataset. Each correlation coefficient is followed by its *P* value. NAS, NAFLD activity score.

The power value was set to 3 for the NAFLD dataset and 8 for the AF dataset to meet the criteria of scale-free topology fit index over 0.85, and the WGCNA network were constructed accordingly (Supplementary Fig. S1). All genes of the NAFLD dataset were divided into 25 modules. We assessed the correlation of each module and age, sex, and five liver pathological features, namely cytological ballooning grade, fibrosis stage, lobular inflammation grade, NAS, and steatosis grade. Similarly, all genes of the AF dataset were divided into 19 modules, and each module was undergone module-trait correlation analysis with AF occurrence. As shown in Fig. 2, the brown (MEbrown in Fig. 2A) and yellow (MEyellow in Fig. 2A) modules from the NAFLD dataset and the black module (MEblack in Fig. 2B) from the AF dataset had top correlation with clinical traits, therefore were chosen as the most relevant modules. GO, KEGG, and REACTOME pathway enrichment analyses of these three modules were performed (Supplementary Fig. S2). The GO results showed that in the NAFLD dataset, the brown module was significantly enriched in processes related to neutrophil-mediated immunity, response to unfolded protein, and neutrophil degranulation. In the same dataset, the yellow module exhibited the highest enrichment in processes related to T cell activation, leukocyte cell–cell adhesion, and lymphocyte proliferation. In the AF dataset, the black module was most enriched in processes related to T cell activation and filamin binding. Across all three key modules, the most significantly enriched GO terms were related to lymphocyte and neutrophil-mediated immunity.

### Construction and enrichment analysis of NAFLD-AF shared gene set (NASGS)

**Figure 3:**
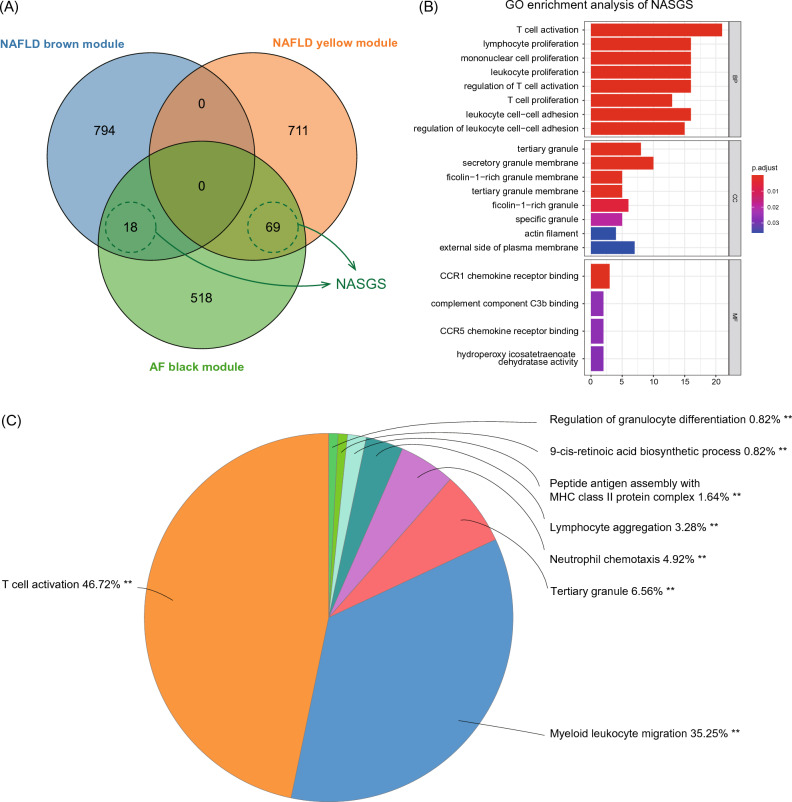
Shared genes and biological pathways of the most relevant NAFLD and AF modules. (**A**) Venn diagram of genes from NAFLD brown module (MEbrown in Fig. 2A), NAFLD yellow module (MEyellow in Fig. 2A), and AF black module (MEblack in Fig. 2B). A NASGS were established combining overlapped NAFLD and AF module genes. (**B**) GO enrichment analysis of NASGS. (**C**) Proportion of each functional group of significantly enriched GO terms by ClueGO software. The size of each part represents the proportion of each GO terms group. GO, gene ontology; NASGS, NAFLD-AF shared gene set. **P < 0.001.

To investigate the potential biological function of shared genes from NAFLD-related modules and AF-related modules, we merged the overlapping genes from two key NAFLD modules, the NAFLD-brown module and NAFLD-yellow module, with one key AF module, the AF-black module. This combined set of genes was referred to as NASGS (Fig. 3A). GO enrichment analysis revealed that NASGS predominantly enriched in T cell-mediated immunity, such as T cell activation (Fig. 3B). To gain a better understanding of the interactions of GO terms, we constructed an integrated GO pathway network from NASGS via ‘ClueGO’ (Supplementary Fig. S3). Consistently, the distribution of functional groups of significantly enriched GO terms suggested that ‘T cell activation’ and ‘myeloid leukocyte migration’ were the top majority clusters (Fig. 3C).

**Figure 4:**
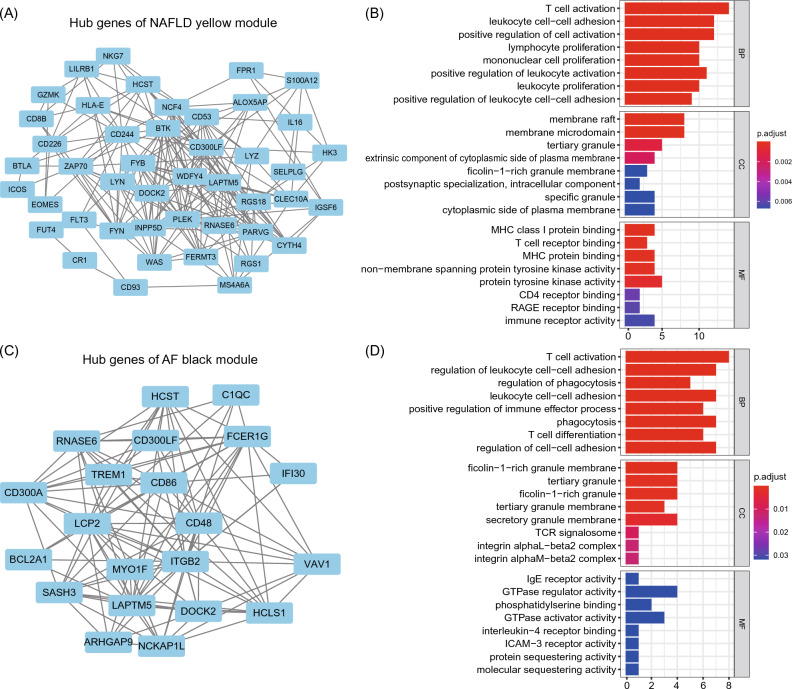
PPI networks and pathway enrichment analysis of highly interconnected clusters from selected NAFLD and AF relevant modules. (**A**) PPI network of hub genes from NAFLD yellow module by MCODE. (**B**) GO enrichment analysis of hub genes from NAFLD yellow module. (**C**) PPI network of hub genes from AF black module by MCODE. (**D**) GO enrichment analysis of hub genes from AF black module. PPI, protein–protein interaction.

Furthermore, we applied GO enrichment analysis to the highly interconnected clusters of NAFLD yellow and brown modules and the AF black module. Results revealed that NAFLD yellow module and AF black module are significantly enriched to the immune process, especially T cell activation (Fig. 4), indicating these two modules are more significantly relevant to the immune process during NAFLD and AF.

To further elucidate the involvement of T cell-mediated immunity in NAFLD and AF, GSE115574, an AF dataset with 59 samples and the NAFLD dataset, were chosen to extract DEGs with the criteria of *P*-value < 0.05. There were 311 genes significantly changed during either NAFLD or AF. GO pathway enrichment analysis of those genes confirmed that immunity, including T cell activation pathway, was involved in NAFLD-AF shared pathophysiological process (Supplementary Fig. S4).

### Immune cell infiltration analysis of AF and NAFLD datasets

**Figure 5:**
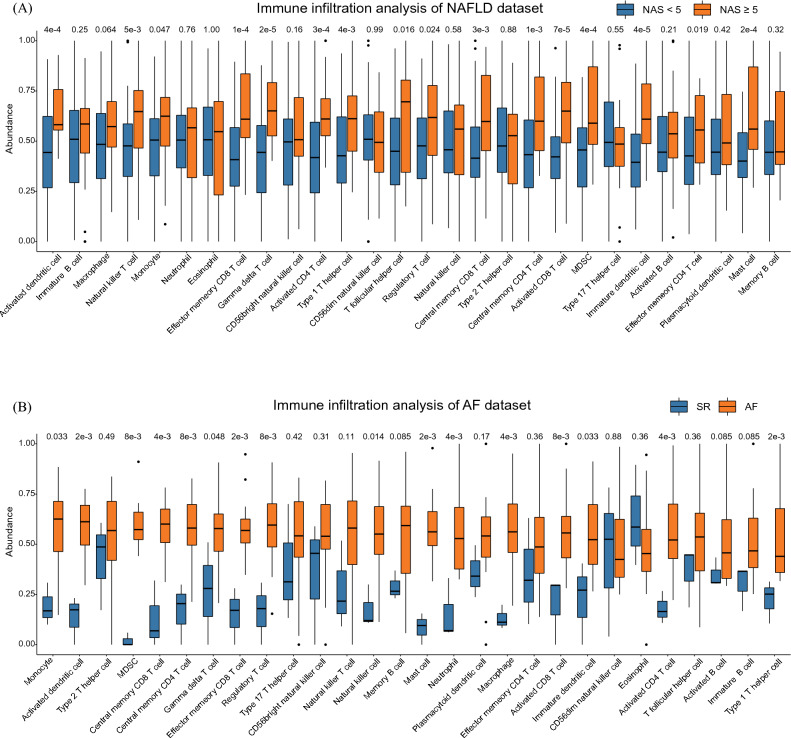
Comparison of immune cells infiltration using single sample gene set enrichment analysis algorithm between (**A**) NAS < 5 and NAS ≥ 5 in NAFLD dataset and (**B**) SR and AF group in AF dataset. NAS, NAFLD activity score.

Enrichment analysis indicated that immune process plays essential roles in both NAFLD and AF. Furthermore, the ssGSEA method was applied to the NAFLD dataset and AF dataset to analyze immune cell abundance in each sample. After dividing liver samples into ‘NAFLD group’ and ‘control group’ by NAS at the cut-off value of 5, we compared the abundance of each immune cell between the two groups. Activated CD4^+^ T cell, activated CD8^+^ T cell, type 1 helper T cell (T_H_1), regulatory T cell, central memory CD4^+^ T cell, central memory CD8^+^ T cell, effector memory CD4^+^ T cell, effector memory CD8^+^ T cell, activated dendritic cell, immature dendritic cell, natural killer T cell, γδT cell, T follicular helper cell, myeloid-derived suppressor cell, monocyte, and mast cell are significantly higher in NAFLD group (*P* < 0.05) (Fig. 5A). In the AF dataset, activated CD4^+^ T cell, activated CD8^+^ T cell, T_H_1, regulatory T cell, central memory CD8^+^ T cell, effector memory CD4^+^ T cell, γδT cell, effector memory CD8^+^ T cell, myeloid-derived suppressor cell, activated dendritic cell, immature dendritic cell, natural killer cell, macrophage, monocyte, mast cell, and neutrophil are significantly higher in AF group (*P* < 0.05) (Fig. 5B).

### Identification of NAGDS from PPI analysis and machine learning

**Figure 6:**
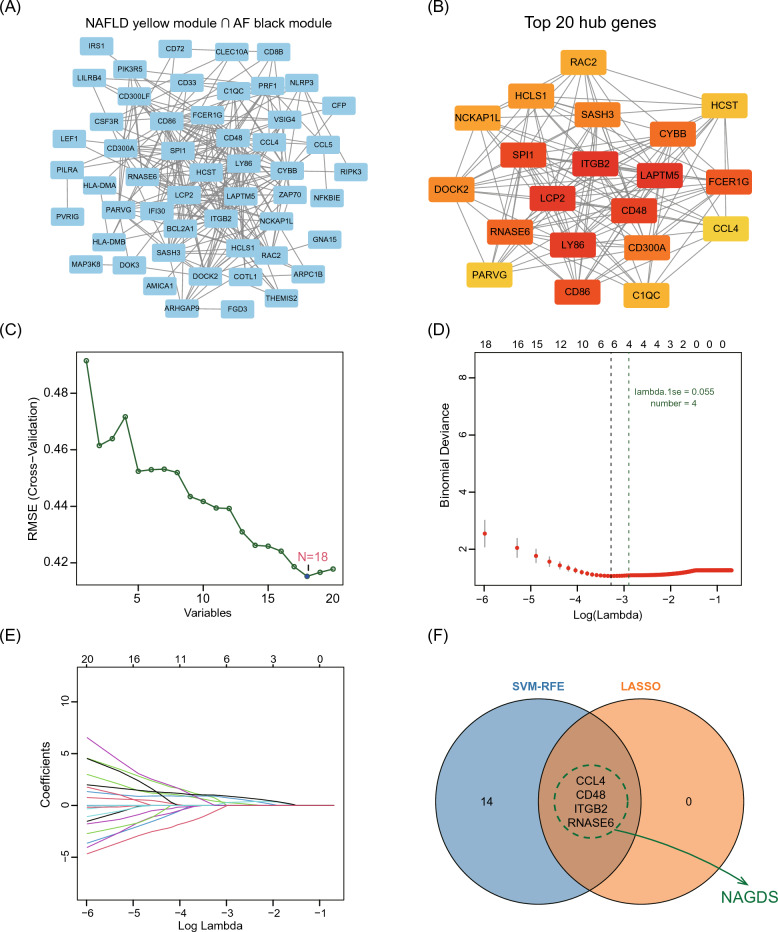
Construction of NAGDS by LASSO regression and SVM-RFE algorithm. (**A**) PPI network of NAFLD-yellow and AF-black modules intersection. (**B**) Top 20 hub genes calculated with MCC of the PPI network. (**C**) SVM-RFE results of the intersected genes. (**D**–**E**) LASSO screening results of the intersected genes. (**F**) Venn diagram shows genes screened by LASSO and SVM-RFE as NAGDS.NAGDS, NAFLD-AF associated gene diagnostic signature.

To establish NAGDS from are modules that most significantly enriched to immune process, the NAFLD yellow module and AF black module were intersected, of which the PPI network were visualized from the STRING database (Fig. 6A). The top 20 hub genes were identified through the maximal clique centrality algorithm (Fig. 6B). SVM-RFE (Fig. 6C) and LASSO regression analysis (Fig. 6D,E) were integrated to increase diagnostic effectiveness while reducing noise information. Four genes were selected by both machine learning algorithms, namely C–C motif chemokine 4 (*CCL4*), CD48 antigen (*CD48*), integrin beta-2 (*ITGB2*), and ribonuclease K6 (*RNASE6*) (Fig. 6F). A regression model derived from machine learning was used to calculate NAGDS score for each sample with the formula: NAGDS score = 0.870 * *CCL*4 + 0.306 * *CD*48 + 0.693 * *ITGB*2 + 0.250 * *RNASE*6 − 6.390.

### Verification of NAGDS in NAFLD and AF datasets

**Figure 7:**
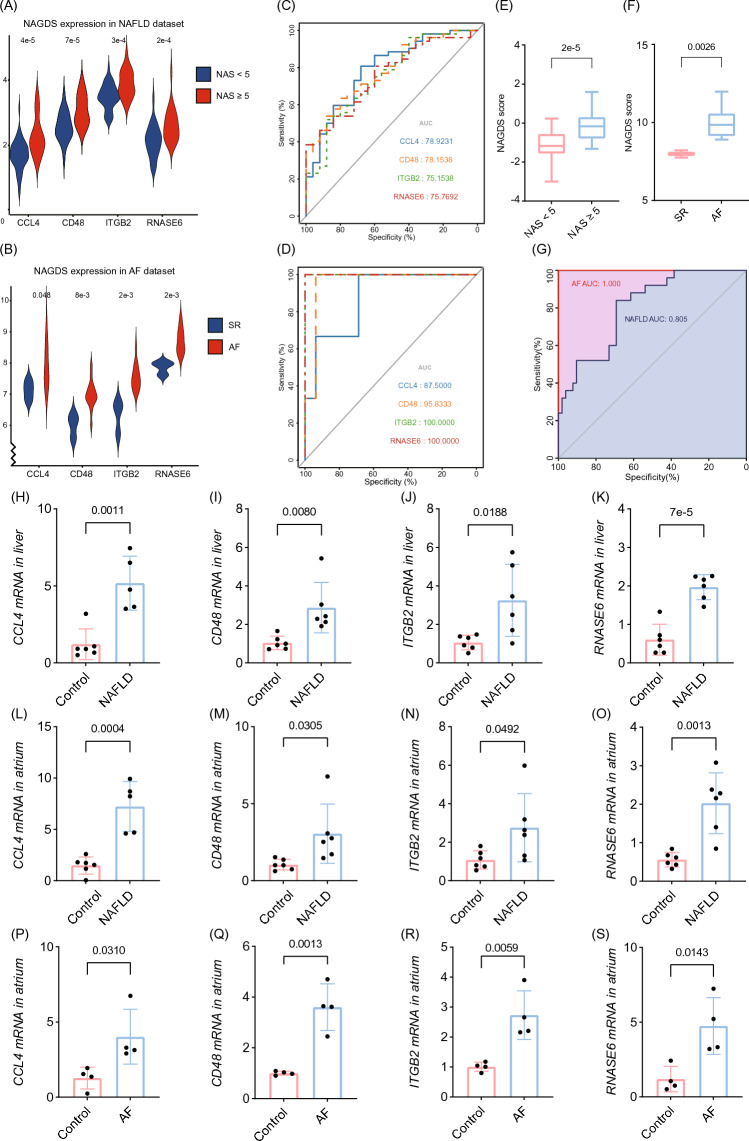
Validation of the NAGDS. (**A**) Expression levels of each NAGDS in NAFLD datasets. (**B**) Expression levels of each NAGDS in AF datasets. (**C**) ROC curve of each NAGDS in NAFLD dataset. (**D**) ROC curve of each NAGDS in AF dataset. (**E**–**F**) Distribution of NAGDS score in NAFLD and AF datasets. (**G**) ROC curve of NAGDS score in NAFLD and AF dataset. (**H**–**K**) The mRNA ex-pression of each NAGDS in liver tissue of NAFLD mice. (**L**–**O**) The mRNA expression of each NAGDS in atrium tissue of NAFLD mice. (**P**–**S**) The mRNA expression of each NAGDS in atrium tissue of AF mice. AUC, Area under the ROC curve.

The expression of each NAGDS was higher than control groups in NAFLD and AF (Fig. 7A,B) dataset (*P* < 0.05). ROC analysis revealed the potential diagnostic performance of each NAGDS reflected by AUC. AUC of *CCL4*, *CD48*, *ITGB2*, and *RNASE6* were 78.92%, 78.15%, 75.15%, and 75.77% in NAFLD dataset (Fig. 7C), and 87.50%, 95.83%, 100%, and 100% in AF dataset (Fig. 7D), indicating the relatively high diagnostic value of the four genes in NAGDS. Next, we evaluated the levels of the NAGDS score and its diagnostic value in the NAFLD and AF datasets. Consistently, the NAGDS score was higher in NAFLD and AF datasets (Fig. 7E,F), and the AUC of the overall NAGDS score was not lower than individual NAGDS (Fig. 7G). To further validate the stability of the NAGDS score, we investigated its ROC curve in two external datasets. The AUC of NAGDS score was 71.4% in GSE63067 NAFLD dataset and 84.0% in GSE14975 AF dataset.

### Verification of NAGDS in liver and atrium of a NAFLD murine model

The expression of each NAGDS was validated in the liver and atrium tissue of NAFLD murine model (n = 12) and the atrium tissue of AF murine model (n = 8) by qRT-PCR. Consistent with results in NAFLD and AF datasets, mRNAs of each NAGDS were significantly upregulated in NAFLD (Fig. 7H–O) and AF (Fig. 7P–S) (*P* < 0.05).

### Biological significance and clinical relevance of NAGDS

**Figure 8:**
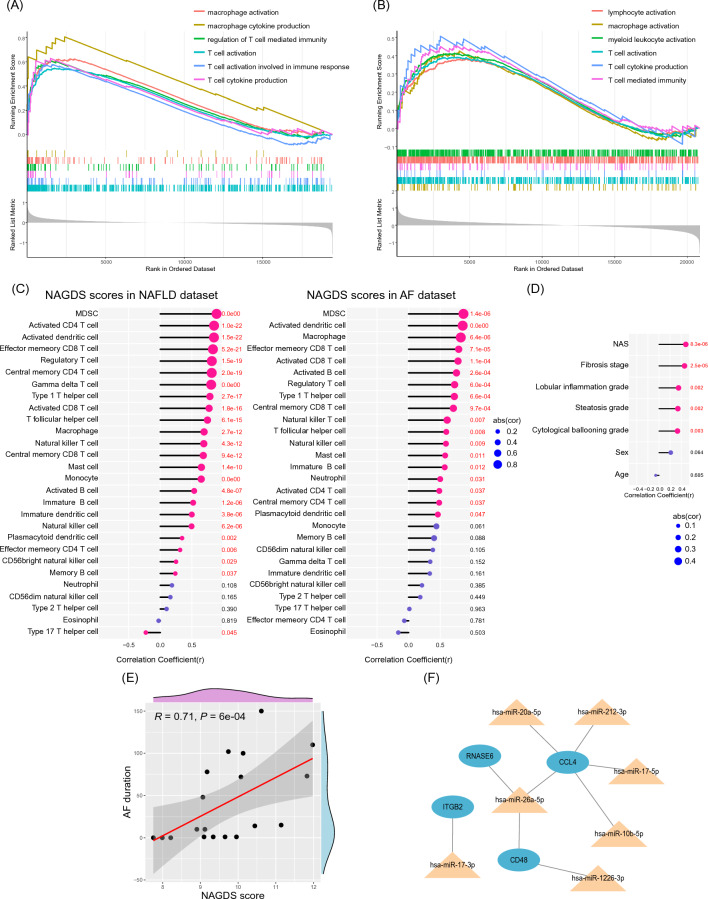
Biological significance and clinical relevance of NAGDS. (**A**) GO GSEA analysis between low and high NAGDS score group in NAFLD dataset. (**B**) GO GSEA analysis between low and high NAGDS score group in AF dataset. (**C**) Correlation analysis between NAGDS scores and immune cell abundance in NAFLD and AF dataset. (**D**) Correlation analysis between NAGDS scores and liver pathological characteristics. (**E**) Correlation analysis between NAGDS scores and AF duration time. (**F**) Therapeutic miRNA-mRNA network derived from NAGDS.

To investigate the biological significance of NAGDS, we regrouped the samples by NAGDS score. GSEA indicated that macrophage- and T cell-related GO terms, such as T cell activation and macrophage activation, were significantly enriched when comparing the high NAGDS score group to the low NAGDS score group in both NAFLD and AF datasets (Fig. 8A,B). Correlation analysis among the NAGDS score and the abundance of 28 immune cells demonstrated that activated T cells and macrophages were significantly positively correlated with NAGDS score in both NAFLD and AF datasets (Fig. 8C). NAFLD and AF datasets were attached with clinical traits, including liver pathological characteristics and AF duration time. Correlation analysis suggested that NAGDS were positively related to liver NAFLD activity score, fibrosis stage, lobular inflammation grade, steatosis grade, and cytological ballooning grade in the NAFLD dataset (Fig. 8D), as well as AF duration time in the AF dataset (Fig. 8E). Correlation analysis of each individual NAGDS and the variables above were shown in detail in Supplementary Fig. S5. We observed that both overall NAGDS score and individual NAGDS were strongly correlated with immune cells such as T cell and macrophage, liver pathological characteristics, and AF duration time. Therefore, we intend to investigate the potential therapeutic regulatory network targeting NAGDS. NAFLD and AF shared a total of 15 miRNAs from the HMDD database, seven of which were found to target NAGDS (Fig. 8F). We proposed that hsa-miR-26a-5p could be of more clinical relevance for the fact that it targeted three of the NAGDS, and played a central role in the posttranscriptional regulatory miRNA-mRNA network.

## Discussion

**Figure 9:**
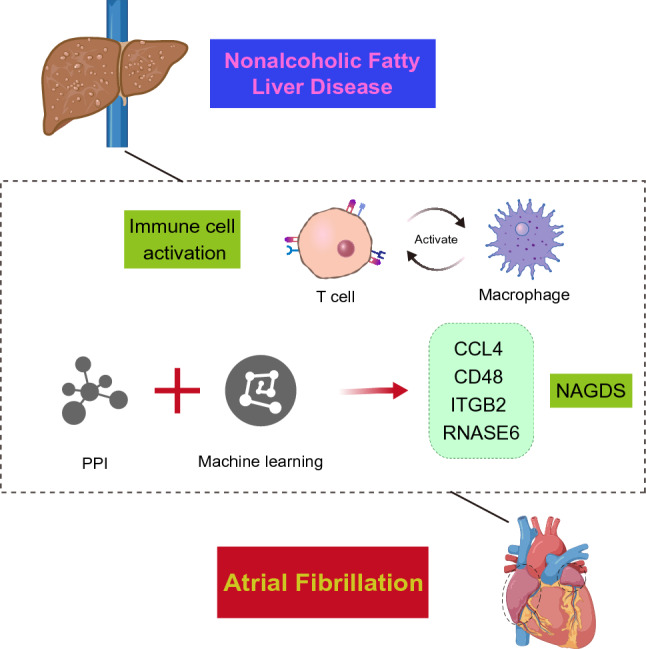
Schematic depicture of T cell and macrophage mediated immunity as shared pathophysiological process of NAFLD and AF.

NAFLD and AF are two different diseases with a high global prevalence, sharing several common risk factors. Many epidemiological evidence, whether cross-sectional or longitudinal, have suggested that NAFLD is associated with an increased risk of prevalent AF^8,31,32^. However, there appears to be limited research exploring the predisposing aspects of AF in NAFLD from the genetic perspective. As shown in Fig. 9, we explored the common mechanisms of NAFLD and AF using bioinformatic methods, such as WGCNA, GO enrichment analysis, and ssGSEA immune cell infiltration, all of which revealed T cell-associated immunity may act as a bridge from NAFLD to AF. Based on machine learning and PPI analysis, we established a NAGDS model composed of four hub genes with precise diagnostic accuracy and the potential to characterize common biological features in NAFLD and AF. The NAGDS model may also alert doctors to the high risks of AF occurrence in NAFLD patients. We also constructed a miRNA-mRNA network targeting the four hub genes, which may be a potential therapy option.

In this study, the gene clusters in AF black module were mainly related to biological processes involved in the immune response, especially T cell activation. Consistent with the above result, we detected that many immune cells were highly infiltrated in the AF atrium, including all kinds of T cell subtypes. T cell is thought to be pivotal in cell-mediated immunity. Previous studies have demonstrated that the number of CD3^+^ T cells was increased in the atrial tissue of AF patients compared to individuals with sinus rhythm^33^. T cells can upregulate hypertrophic genes and induce atrial hypertrophy during AF^34^. Distinct helper T(T_H_) cell subsets, differentiated from naive CD4^+^ T cell, is mainly characterized by the cytokines they produce. T_H_1 cells can produce IFN-γ, which was proved to be markedly elevated in plasma of AF patients^35,36^. IFN-γ exerts various biological effects that are predicted to promote atrial remodeling through macrophages by stimulating cytokines secretion. A recent study found that IL-17A, produced by the type 17 T helper cell (T_H_17, differentiated from CD4^+^ T cell in response to inflammatory stimuli), leads to the development of AF by promoting inflammation and cardiac fibrosis^37^. These results suggested a link between immune response and AF, and T cell activation may be pivotal in the immunological pathogenesis of AF.

T cell activation and its associated immune response are believed to be essential features in NAFLD, consistent with our GO analysis result of the NAFLD yellow module. Our ssGSEA results also showed that the number of CD4^+^ and CD8^+^ T cells was elevated in the liver tissue of NAFLD patients (Fig. 5). In different experimental models of NASH, CD4^+^ and CD8^+^ T cells activation and infiltration in the liver are evident and closely related to worsening liver injury and inflammation^37–42^. IFN-γ-producing T_H_1 cells and IL-17-producing T_H_17 cells are recruited to the liver at the onset of steatohepatitis^43–46^. Luo, X. Y. et al. found that the steatohepatitis and fibrosis in IFN-γ deficient mice are less severe than wild type mice when fed with a methionine-choline-deficient (MCD) high-fat diet^47^. T_H_17 cells can produce IL-17 and, to a lesser extent, IL-21, IL-22, IFN-γ, and TNF^46^. Several studies have shown that the activated IL-17 axis contributes to the development and progression of NAFLD to steatohepatitis^38,48^. In addition, cytotoxic CD8^+^ T cells are also increased and activated in the development of NAFLD^38,39,43^, which is associated with IFN-γ and LIGHT (Homologous to Lymphotoxin, belongs to the TNF superfamily of proinflammatory molecules) production^39^. The network of cytokines produced by T_H_1, T_H_17, and CD8^+^ cells not only has a direct proinflammatory effect but also strongly stimulates M1 hepatic macrophages, which are thought to be significant instigators of liver damage in NAFLD.

AF has a high prevalence in NAFLD, suggesting that some predisposing factors in NAFLD may trigger the onset and development of AF. In our WGCNA analysis, whether in the NAFLD or AF, the function of gene clusters in both highly related modules was mainly enriched in adaptive immunity, especially T cell activation (Supplementary Fig. S2). The function of NASGS in positivity-related modules of NAFLD and AF was also mainly enriched in T cell activation. Thus, activated T cell-mediated adaptive immunity may be a common feature in the pathophysiology of NAFLD and AF. In NAFLD, as commonly acknowledged, oxidative stress, together with metabolic stress and ER stress, leads to cell death and the release of danger-associated molecular patterns (DAMPs), initiating innate immune responses and further inducing hepatic adaptive immunity^49^. CD4^+^ and CD8^+^ T cells actively participate in this complex process and release proinflammatory cytokines and chemokines, which can potently stimulate the M1 hepatic macrophage, leading to liver inflammation^50,51^.

Similarly, our analysis shows that the hub genes of yellow modules in NAFLD also include several proinflammatory mediators, such as TNF, CCL3, CCL4, IL-18, etc. Importantly, these inflammatory-related mediators and DAMPs can circulate from the liver into the systemic bloodstream, potentially fostering systemic inflammation^52^, which could impact extrahepatic tissues such as the heart. Once the DAMPs and inflammation mediators reach atrial tissue, they can contribute to local inflammation and its associated immune response, such as the activation of T cells, B cells, and macrophages^49^, which is consistent with our function enrichment analysis in AF black module (Fig. 5).

Inflammation and its associated immune response are involved in the initiation and maintenance of AF. Many inflammatory cytokines, including TNF-α, IFN-γ, IL-1β, IL-2, IL-6, and IL-18, have found te be elevated in AF patients^35,36,53,54^. Moreover, several proinflammatory cytokines, such as TNF-α, IL-1β, and IL-17, have been shown to induce cardiac arrhythmias in various animal models^55–57^. Mechanistically, increased proinflammatory mediators observed in NAFLD can potentially cause structural and electrical atrial remodeling, promoting the development of AF. T_H_1 cells, for instance, can secrete IFN-γ, which provides a potent stimulus for atrial macrophages. The TNF-α secreted by activated macrophages is thought to be more than an inflammatory mediator in AF patients but also inflicts proarrhythmic remodeling. Firstly, TNF-α had a pathological effect on atrial fibrosis and changed the expression or distribution of Cx40 (connexin-40) and Cx43^58,59^, leading to heterogeneous conduction. Secondly, TNF-α has been shown to induce Ca^2+^ handling dysfunction by decreasing the expression of SERCA (sarco-endoplasmic reticulum Ca^2+^ ATPase) 2α^60^. Thirdly, TNF-α enhances myocardial apoptosis and myolysis, which are associated with atrial dilatation and conduction heterogeneity^61^. Once AF initiates, it may in turn lead to calcium overload in atrial myocytes, resulting in cell death, DAMPs release, and triggering a subsequent low-grade inflammatory response activation. This phenomenon is commonly referred to as 'AF begets AF'. Based on our analysis results and existing theories, inflammation and immune response play an irreplaceable role in the mechanisms of AF secondary to NAFLD, especially T cell-mediated adaptive immunity.

To find novel diagnostic targets with significant biological functions and diagnostic applications, we used machine learning to further screen NAFLD-AF-related genes from the hub genes in immune-related gene modules. Four overlapping genes (CCL4, CD48, ITGB2, and RNASE6) were selected to establish the NAGDS. According to the bioinformatics analysis result and previous research, it's evident that these four hub genes interact with one another and are intimately associated with immune responses. CCL4 (previously known as macrophage inflammatory protein (MIP)-1beta, MIP-1β) is an important proinflammatory chemokine for the recruitment of T cells and macrophages^62^. CCL4 has been reported to be elevated in both NAFLD and AF patients^63,64^, a finding consistent with our results (Fig. 7). CCL4 can interact with its specific receptor, CCR5, which can promote fibrosis by activating hepatic stellate cells and recruiting macrophages^65,66^. These facts further indicate that CCL4 may promote atrial remodeling through macrophages by stimulating cytokines secretion. CD48 is a lipid-anchored protein expressed on the membrane surface of all antigen-presenting cells and T cells. It participates in T-cell signaling transduction by interacting with CD2, a key player in T-cell activation, making CD48 an important component of T-cell activation pathways^67^. ITGB2, also known as CD18, is a receptor located on the surface of T lymphocytes, neutrophils, and monocytes^68,69^. It plays a role in facilitating their adhesion, transmigration, and infiltration into injured tissue^70^. In the context of early NASH, CD18 deficiency has been shown to limit hepatic injury by inhibiting the activation and infiltration of immune cells in MCD-fed mice^71^. Friedrichs et al. showed that CD11b/CD18 mediated polymorphonuclear neutrophils infiltration contributed to the atrial fibrosis, which increased the susceptibility of AF in angiotensin II treated mice^72^. Given these findings, CD18 can conceivably play a significant role in the AF secondary to NAFLD by promoting the infiltration of immune cells. RNASE6 belongs to the secreted protein of the Ribonuclease A superfamily and is associated with many physiological functions, including immunity, cytotoxicity, and angiogenesis^73–75^. There have been reports linking promoter methylation of Rnase6 to processes like cell proliferation, migration, oxidative stress, and inflammation in mouse aortic smooth muscle cells^76^. However, the precise role of RNASE6 in both NAFLD and AF is poorly studied and needs further investigation.

The current diagnosis of NAFLD relies on liver enzymes and imaging methods^77^, which do not provide doctors with insights into the elevated risks of AF occurrence in NAFLD patients. Thus, we established a diagnostic model consisting of the above four key genes (NAGDS), which showed higher accuracy and stability, and might cover the above shortage of traditional diagnosis methods in the following ways: (a) NAFLD patients diagnosed by the NAGDS model may also have co-existed AF; (b) A high NAGDS score is indicative of more severe pathological lesions and a increased risk of AF persistence in NAFLD-AF patients (Fig. 8); (c) for NAFLD-AF patients, a high NAGDS score signifies a heightened activation of T cell and macrophage, potentially indicating unfavorable biological modifications that require closer monitoring or prompt therapeutic intervention. Overall, NAGDS not only aids in the precise diagnosis of NAFLD but also serves as a reminder to healthcare professionals that diagnosed patients may also have concurrent AF.

The function of post-transcriptional regulation of miRNA has been widely demonstrated to be associated with the onset of various diseases^78^. Therefore, we took advantage of the HMDD database and the miRTarbase to construct the common miRNAs-shared genes network targeting the NAGDS. Among these miRNAs, miR-26a targeted the most genes in NAGDS, including CCL4, CD48, and RNASE6. MiR-26a is broadly expressed at high levels in human tissues but was found reduced in the liver of NAFLD patients compared with non-steatosis individuals^79–82^. MiR-26a can increase insulin sensitivity and attenuate obesity-related metabolic dysfunction in the liver^80^. Mounting evidence has shown that miR-26a modulated immunological functions in different experimental mouse models, such as promoting regulatory T cell expansion^83–85^. A recent study also demonstrated that miR-26a might prevent NAFLD via an immune-regulatory axis consisting of IL-6 and IL-17^86^. Thus, it is possible that the multifunctional roles of miR-26a collectively contribute to attenuating the development of NAFLD. The expression of miR-26 was also reported to be decreased in atrial samples from AF patients and animals, and this downregulation may promote AF-related electrical remodeling by controlling the expression of KCNJ2^87^. Our study also found that the NAGDS genes targeted by miR-26a are closely related to the development of AF. Taken together, miR-26a might be an important potential target for the treatment of NAFLD and AF.

There are limitations in our present study. Firstly, the establishment and authentication of the NAGDS model were carried out on publicly available datasets with a limited number of samples; a larger validation cohort and identification of optimal cutoff values are needed before translated to clinical practice. Besides, observation of AF induction rate in HFD-induced NAFLD murine and its correlation with NAGDS expression would provide more direct evidence of causal relationship between NAFLD and AF. However, we couldn’t detect AF inducibility in NAFLD mice due to the lack of murine electrophysiological device. Finally, a further investigation of the biological interaction between miR-26a and its target genes in an experimental model remains to be carried out.

## Conclusions

In summary, our work proposed an immune-regulatory network between NAFLD and AF, firstly revealing the T cell activation mediated immune response in NAFLD might be an essential predisposed factor for AF, and establishing the NAGDS model, which could be used to diagnose NAFLD and AF accurately. These NAGDS genes may also be potential targets for immunoregulatory therapy.

### Supplementary Information

Supplementary Figures.

Supplementary Tables.

Supplementary Table 3.

## Data Availability

The datasets GSE41177, GSE130970, GSE115574, GSE14975, and GSE63067 for this study can be found in online repositories (http://www.ncbi.nlm.nih.gov/geo). Basic information of the datasets was listed in detail in Supplementary Table S2.
