# Supplementary material for: Exploring immune related gene signatures and mechanisms linking non alcoholic fatty liver disease to atrial fibrillation through transcriptome data analysis
Source: Sci Rep. 2023 Oct 16;13:17548. doi: 10.1038/s41598-023-44884-z (PMC10579333; doi:10.1038/s41598-023-44884-z)
Supplement: Supplementary file 1 — Supplementary Figures. [file 41598_2023_44884_MOESM1_ESM.docx]

Supplementary Material

# Supplementary Figures


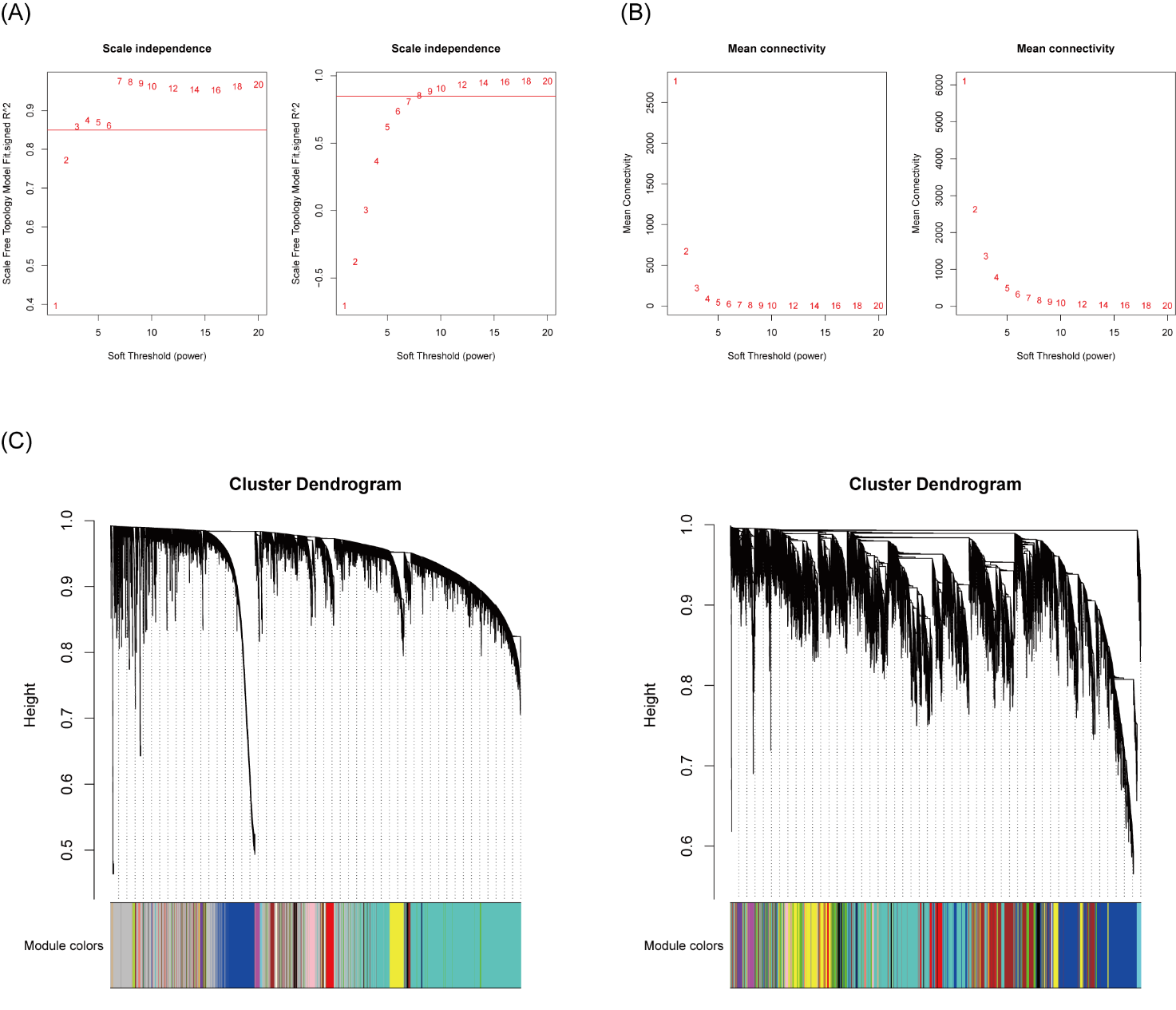


**Figure S1 Weighted gene co-expression network analysis (WGCNA).** (A) Scale independence for various soft-thresholding powers in NAFLD dataset (left) and AF dataset (right). (B) Mean connectivity for various soft-thresholding powers in NAFLD dataset (left) and AF dataset (right). (C) Cluster dendrogram of genes based on a dissimilarity measure in NAFLD dataset. (D) Cluster dendrogram of genes based on a dissimilarity measure in AF dataset.


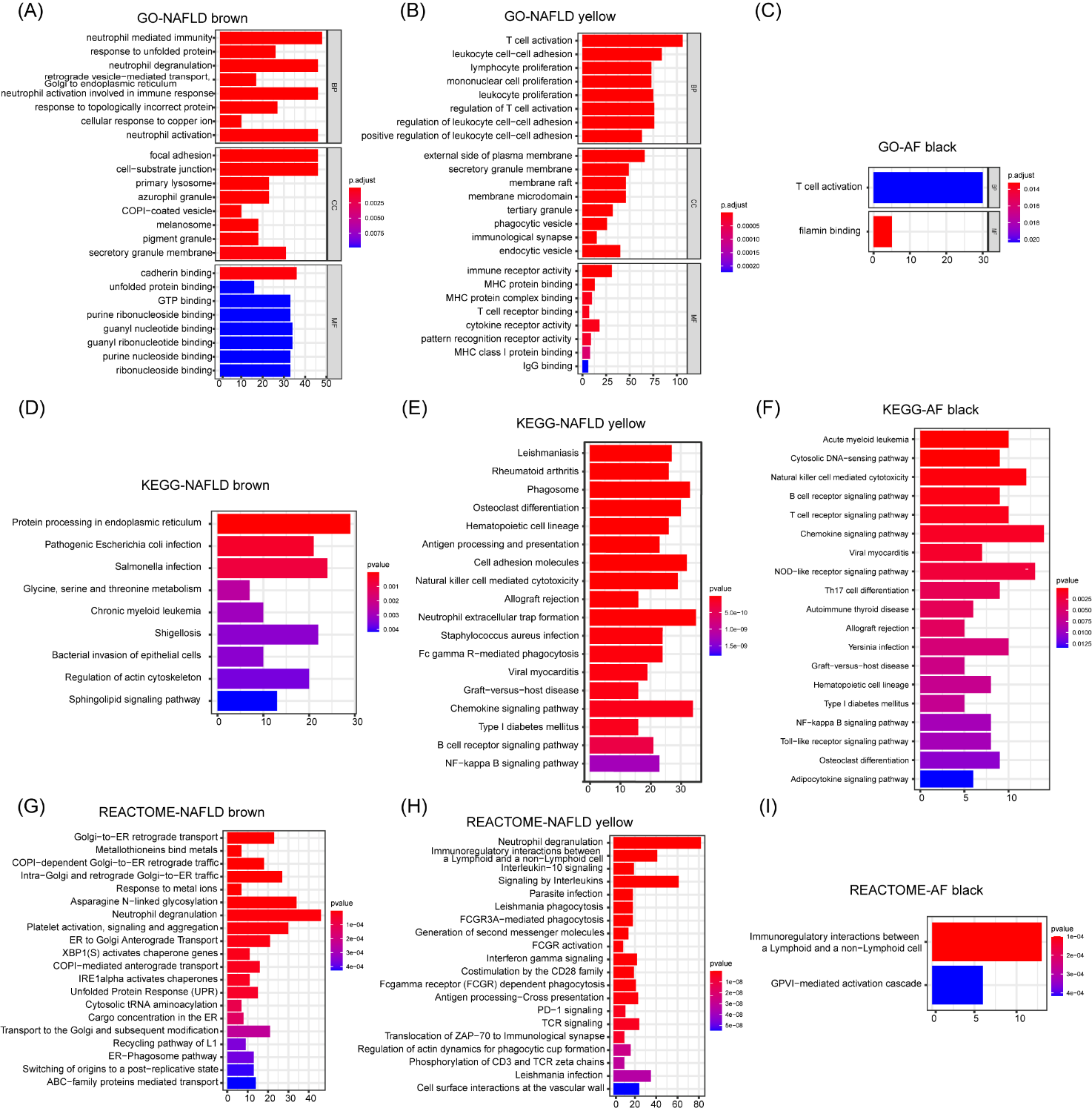


**Figure S2 Pathway enrichment analysis of most relevant NAFLD and AF modules.** (A) GO enrichment analysis of NAFLD brown module. (B) GO enrichment analysis of NAFLD yellow module. (C) GO enrichment analysis of AF black module. (D) KEGG enrichment analysis of NAFLD brown module. (E) KEGG enrichment analysis of NAFLD yellow module. (F) KEGG enrichment analysis of AF black module. (G) REACTOME enrichment analysis of NAFLD brown module. (H) REACTOME enrichment analysis of NAFLD yellow module. (I) REACTOME enrichment analysis of AF black module.


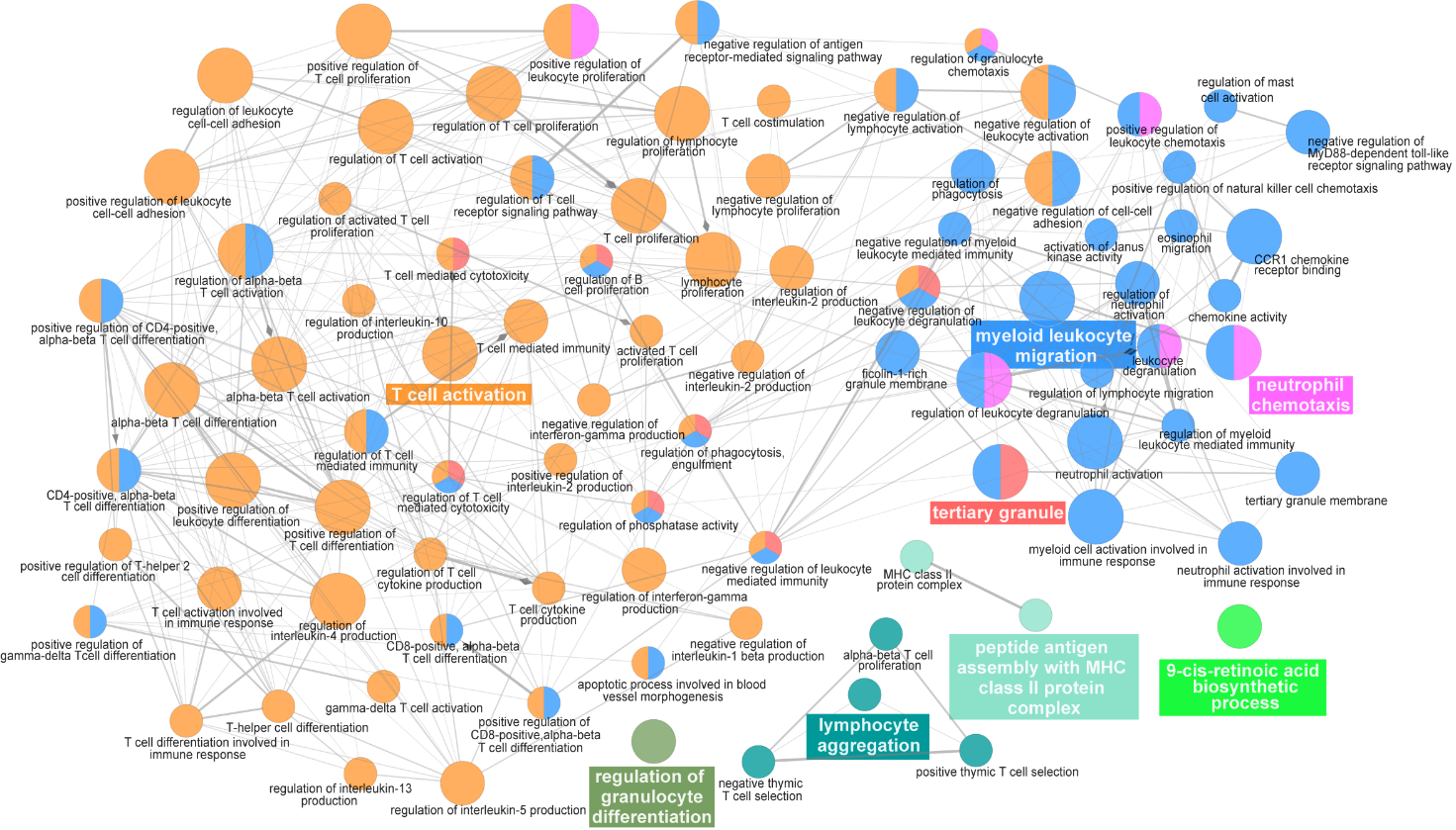


**Figure S3** **Functional group network of GO terms derived from NASGS by ClueGO software.** Node side reflected term enrichment significance, and edge thickness represented the kappa score of the connection.


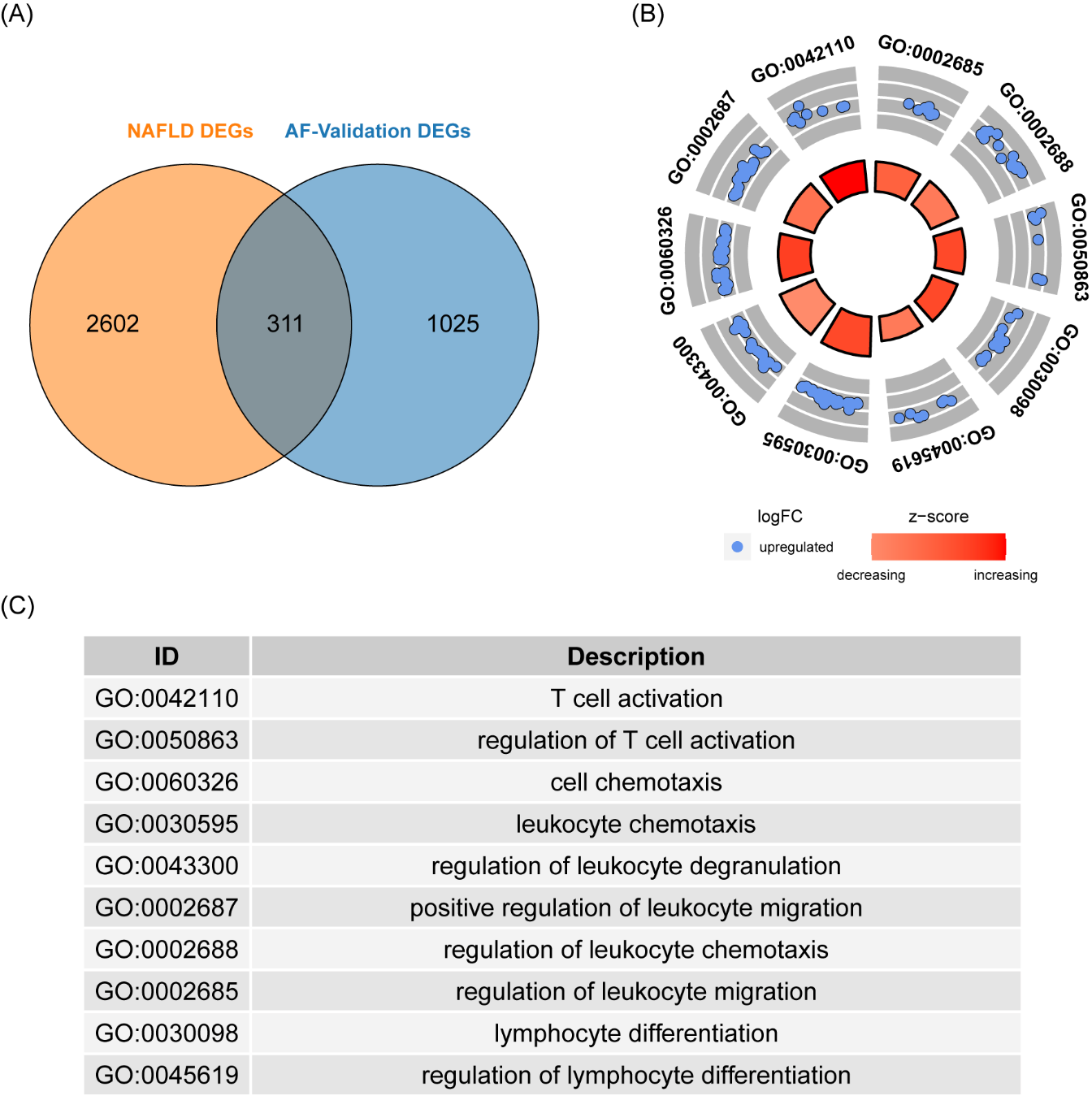


**Figure S4 GO enrichment analysis of overlapped differentially expressed genes (DEGs) from NAFLD dataset and AF validation dataset.** (A) Venn diagram of DEGs. (B-C) GO enrichment of common upregulated DEGs.


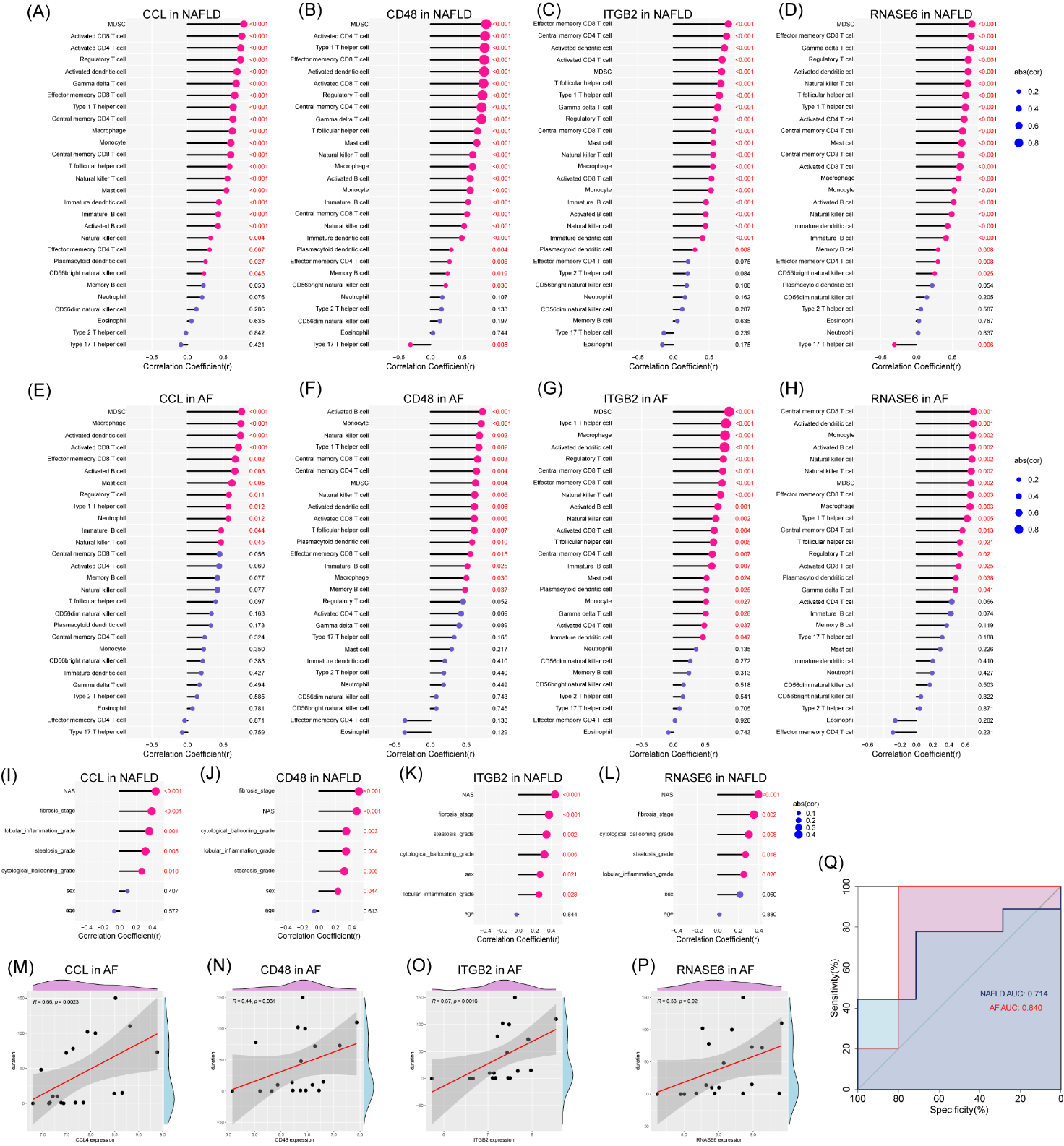


**Figure S5 Correlation analysis between each NAGDS and immune cell abundance, liver pathological characteristics, and AF duration time.** (A-D) Spearman correlation between expression of each NAGDS and immune cell abundance in NAFLD dataset. (E-H) Spearman correlation between expression of each NAGDS and immune cell abundance in AF dataset. (I-L) Spearman correlation between expression of each NAGDS and liver pathological characteristics in NAFLD dataset. (M-P) Spearman correlation between expression of each NAGDS and AF duration time in AF dataset. (Q) ROC curve of NAGDS score in GSE63067 NAFLD dataset and GSE14975 AF dataset.
