# Supplementary material for: Exploring immune related gene signatures and mechanisms linking non alcoholic fatty liver disease to atrial fibrillation through transcriptome data analysis
Source: Sci Rep. 2023 Oct 16;13:17548. doi: 10.1038/s41598-023-44884-z (PMC10579333; doi:10.1038/s41598-023-44884-z)
Supplement: Supplementary file 2 — Supplementary Tables. [file 41598_2023_44884_MOESM2_ESM.docx]

Supplementary Material

# Supplementary Tables

**Supplementary Table 1** Sequences of primers used for real-time PCR.

| Species | Genes | Sequences |
| --- | --- | --- |
| Mouse | β-actin | Forward: 5’-GGCTGTATTCCCCTCCATCG-3’  Reverse: 5’-CCAGTTGGTAACAATGCCATGT-3’ |
| Mouse | CCL4 | Forward: 5’-TTCCTGCTGTTTCTCTTACACCT-3’  Reverse: 5’-CTGTCTGCCTCTTTTGGTCAG-3’ |
| Mouse | CD48 | Forward: 5’-CCCAAGCCTTCCATAGAAATCAA-3’  Reverse: 5’-CCAAGTATAGTCAACATGCTGGT-3’ |
| Mouse | ITGB2 | Forward:5’-CAGGAATGCACCAAGTACAAAGT-3’  Reverse: 5’-CCTGGTCCAGTGAAGTTCAGC-3’ |
| Mouse | Rnase6 | Forward: 5’-CTGTGGGAGCCGATGTATCTA-3’  Reverse: 5’-TTGCATGGTTGACGACTTGTC-3’ |

**Supplementary Table 2** **Baseline information of the datasets**

| GEO accession | Disease | Samples (n) | Platform | Tissue | |
| --- | --- | --- | --- | --- | --- |
| GSE41177 | Persistent AF | 19 | GPL570 | | Left atrial appendage |
| GSE130970 | NAFLD | 78 | GPL16791 | | Liver |
| GSE115574 | Permanent AF | 59 | GPL570 | | Atrial tissue |
| GSE14975 | AF | 10 | GPL570 | | Left atrial tissue |
| GSE63067 | NAFLD | 18 | GPL570 | | Liver |
